# Supplementary material for: A reversible phospho-switch mediated by ULK1 regulates the activity of autophagy protease ATG4B
Source: Nat Commun. 2017 Aug 18;8:294. doi: 10.1038/s41467-017-00303-2 (PMC5562857; doi:10.1038/s41467-017-00303-2)
Supplement: Supplementary file 3 — Supplementary Information [file 41467_2017_303_MOESM3_ESM.pdf]

Description of Supplementary Files

Title: Supplementary Information

Description: Supplementary Figures

Title: Peer Review File

Title: Supplementary Data 1
Description: Raw values of phosphatase cDNA expression screen using the luciferase release assay.

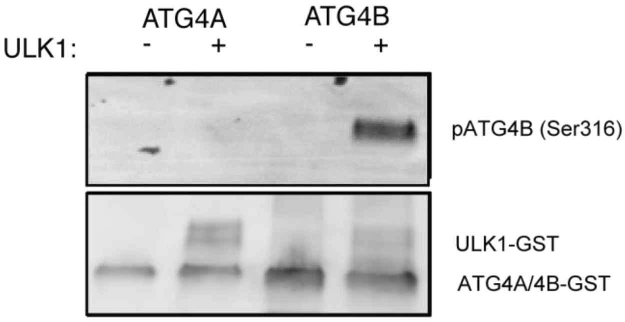

Supplementary Figure 1: Recombinant human ATG4B but not ATG4A is detected with pATG4B(Ser 316) antibody in an *in vitro* ULK1 phosphorylation assay. Recombinant GST-tagged ATG4A and ATG4B were incubated at 0.5 μg with 0.1 μg ULK1 for 30 min at 37°C and subjected to western blotting using anti-pATG4B(Ser 316) and anti GST antibody. The upper panel shows that phosphorylation of ATG4B occurs in the presence of ULK1, but no band is detected for ATG4A. The lower panel shows that all GST-tagged proteins (ATG4A, ATG4B and ULK1) were present in the reaction mixture.

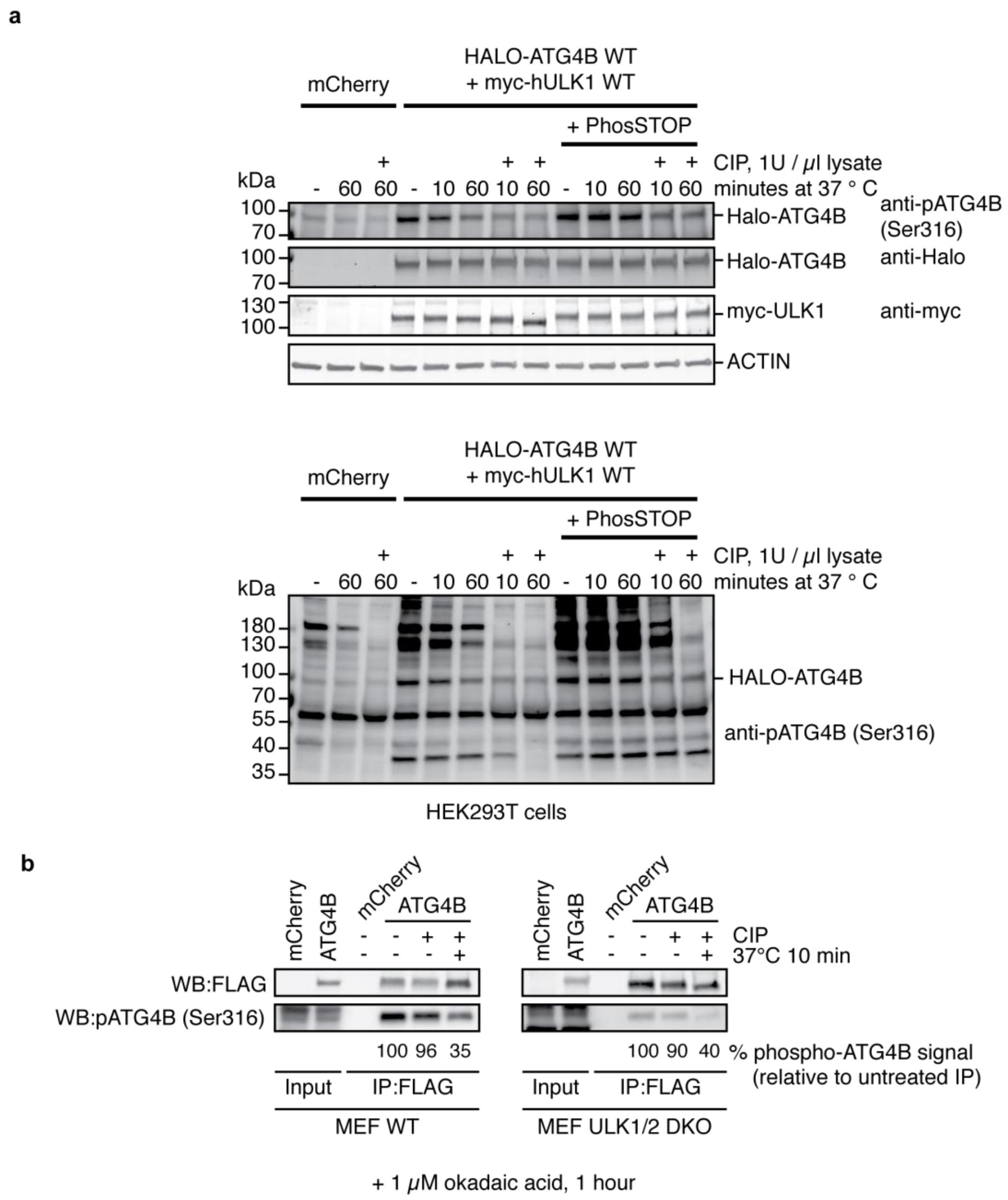

Supplementary Figure 2: Validation of the pATG4B(Ser316) antibody using phosphatase treatment of cell extracts to demonstrate phosphorylation-dependency.

(**a**) HEK293T cells were co-transfected with Halo-ATG4B and myc-hULK1 or transfected with mCherry alone as a control. After 24 hours, cells were resuspended in PBS, and cell suspensions were divided into equal volumes before being centrifuged at 300 x g for 5 minutes and lysed on ice in 1% NP-40 lysis buffer (with protease

inhibitor) with or without phosphatase inhibitor (PhosSTOP). Lysates were cleared by centrifugation before being subject to phosphatase treatment (see methods). Halo-ATG4B signal detected by anti-pATG4B(Ser316) was reduced by phosphatase treatment, and this effect was diminished in the presence of phosphatase inhibitor. Lower panel shows full blot detected by anti-pATG4B(Ser316), with a number of additional bands detected by the antibody also showing a similar phosphorylation dependency, and the specific band of Halo-ATG4B is indicated.

(**b**) Immunoprecipitated samples from experiment in Figure 2e were subject to phosphatase treatment and blotted using anti pATG4B(Ser316) to assess 3xFLAG-ATG4B phosphorylation and anti FLAG as a control for total 3xFLAG-ATG4B. The phosphorylated ATG4B signal was determined as a % of untreated IP using densitometry, with pATG4B(Ser316) signal normalised against total ATG4B signal for the same band. In both WT and ULK1/2 DKO MEFs, phosphatase treatment reduced the signal for 3xFLAG-ATG4B using the pATG4B(Ser316) antibody.

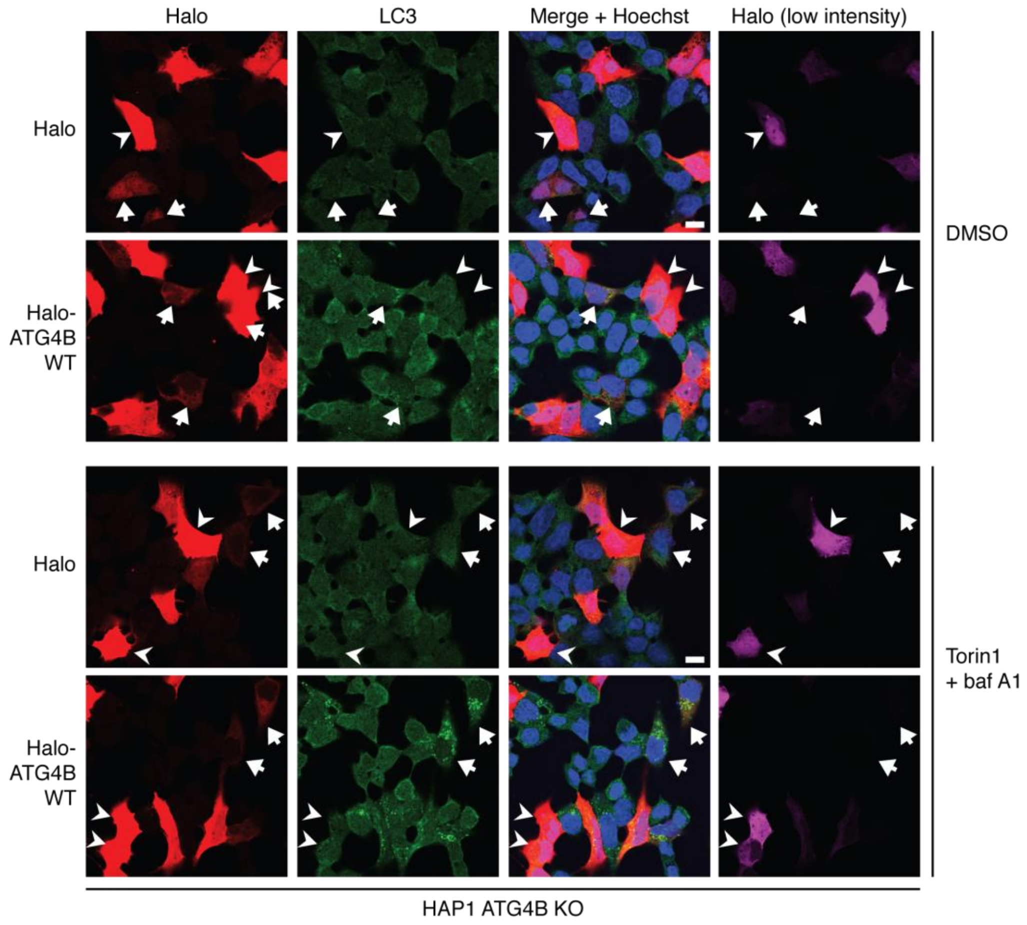

Supplementary Figure 3: Control images for experiment shown in Figure 4c. The images were acquired in the same experiment as shown in Figure 4c. Cells treated with DMSO show reduced numbers of LC3-positive autophagosomes, confirming that LC3 puncta formation is dependent on treatment with Torin1 + bafilomycin A1 in ATG4B KO cells rescued with Halo-ATG4B. The right panels (in magenta) show the Halo staining acquired at a reduced laser intensity that was used to threshold high-expressing cells without saturation of the image, in order to discriminate them from low-expressing cells. Under these settings, low- expressing cells are not visible by eye.

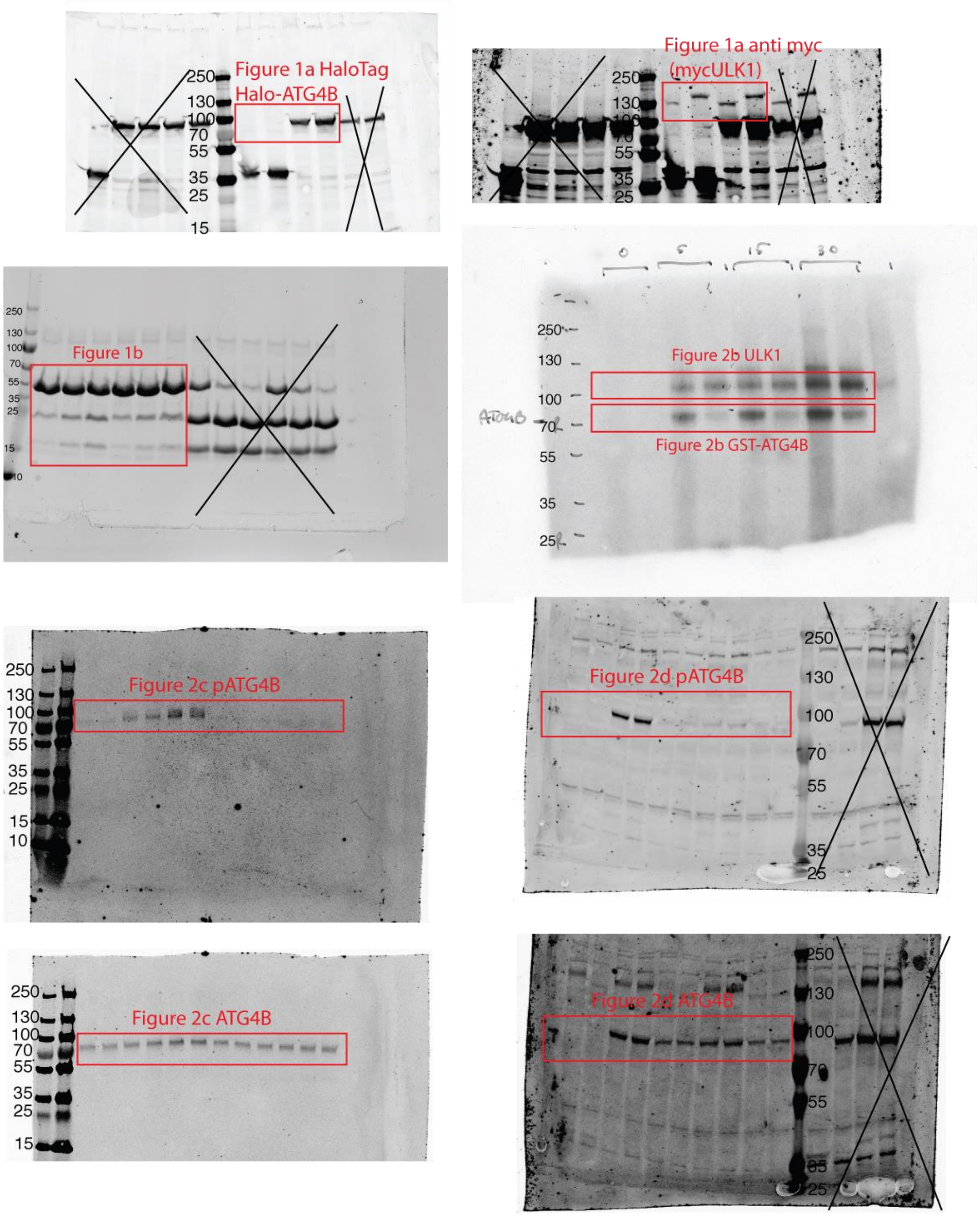

Supplementary Figure 4: Uncropped gels and blots for key figures in the manuscript. The red boxes indicate the section used in the Figures. (continued on next pages)

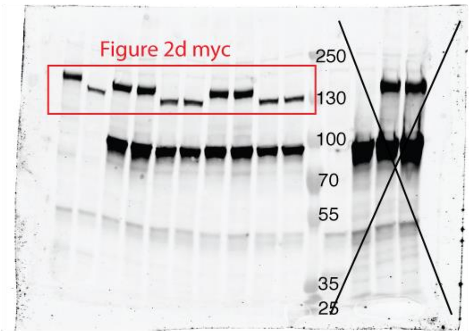
Figure 2d myc
250
130
100
70
55
35
25

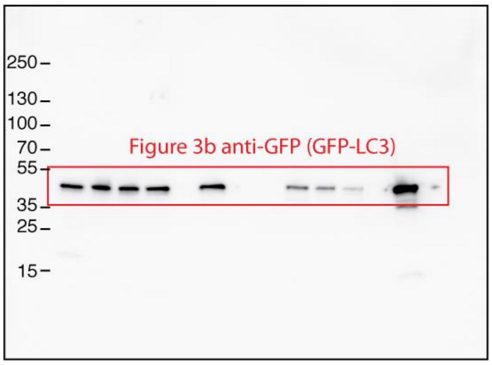
Figure 3b anti-GFP (GFP-LC3)
250
130
100
70
55
35
25
15

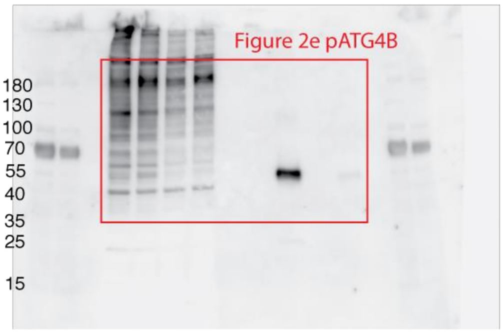
Figure 2e pATG4B
180
130
100
70
55
40
35
25
15

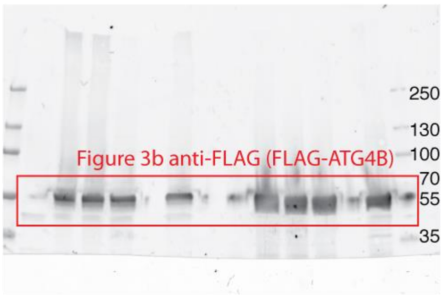
Figure 3b anti-FLAG (FLAG-ATG4B)
250
130
100
70
55
35

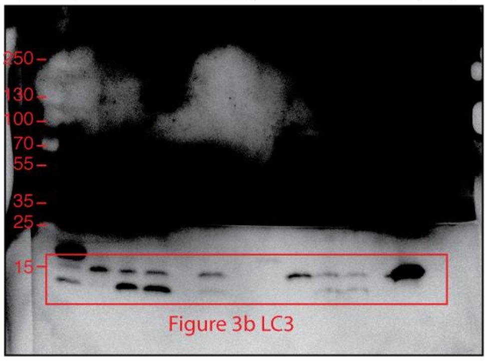
250
130
100
70
55
35
25
15
Figure 3b LC3

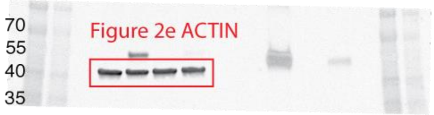
Figure 2e ACTIN
70
55
40
35

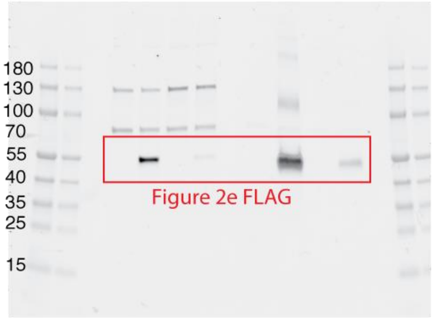
180
130
100
70
55
40
35
25
15
Figure 2e FLAG

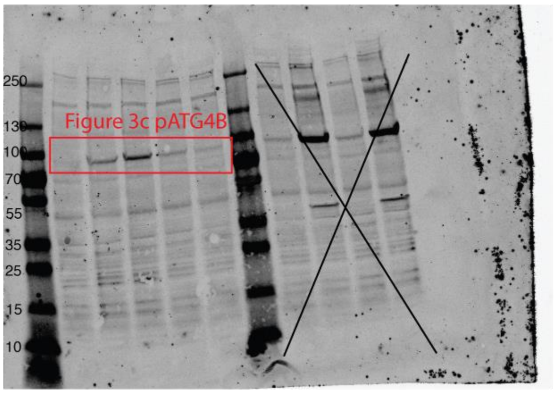
250
130
100
70
55
35
25
15
10
Figure 3c pATG4B

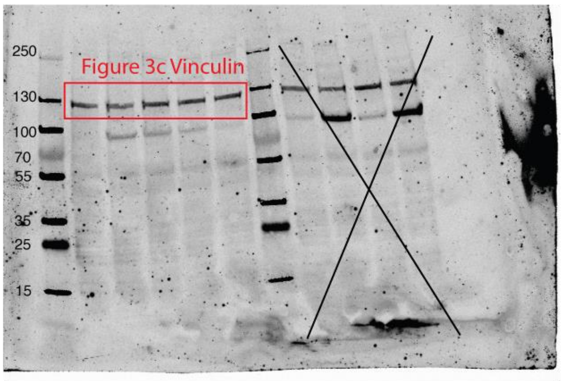
Figure 3c Vinculin
250
130
100
70
55
35
25
15

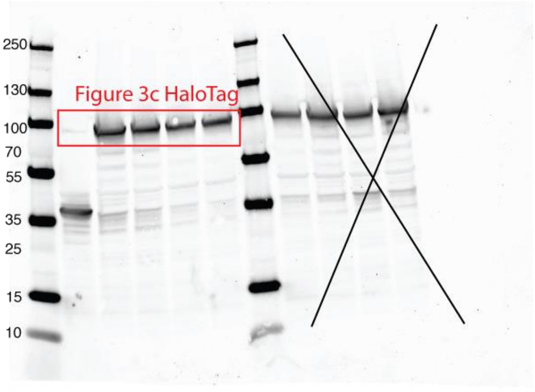
Figure 3c HaloTag
250
130
100
70
55
35
25
15
10

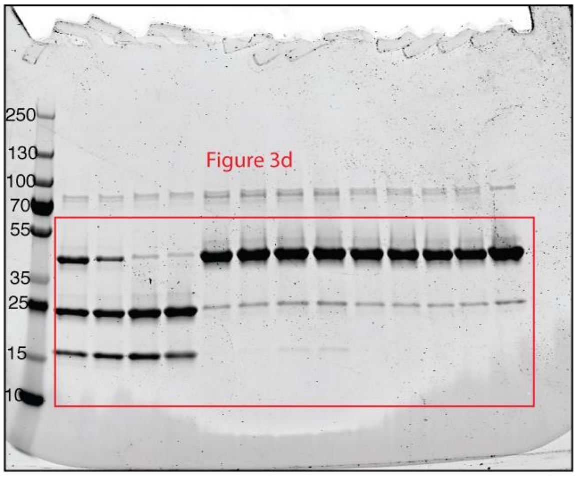
Figure 3d
250
130
100
70
55
35
25
15
10

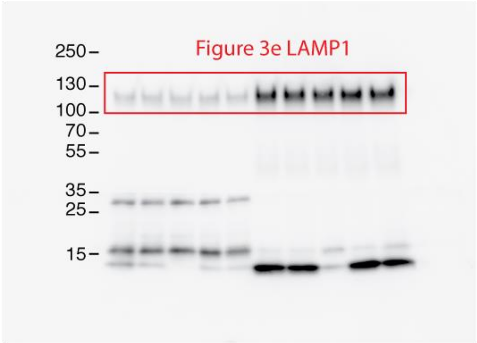
Figure 3e LAMP1
250
130
100
70
55
35
25
15

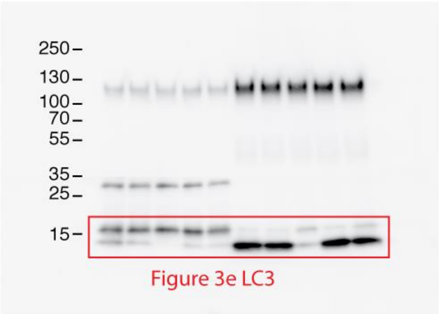
250
130
100
70
55
35
25
15
Figure 3e LC3

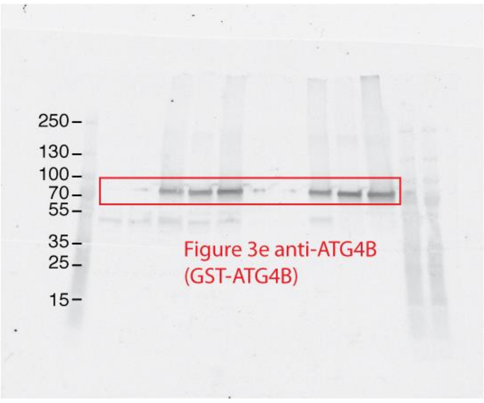
250
130
100
70
55
35
25
15
Figure 3e anti-ATG4B
(GST-ATG4B)

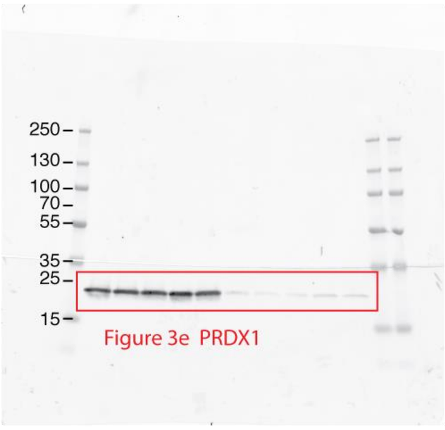
250
130
100
70
55
35
25
15
Figure 3e PRDX1

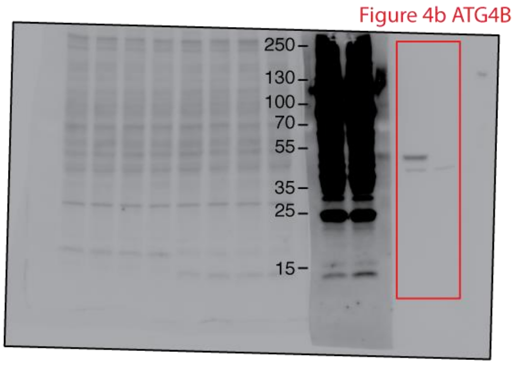
Figure 4b ATG4B
250
130
100
70
55
35
25
15

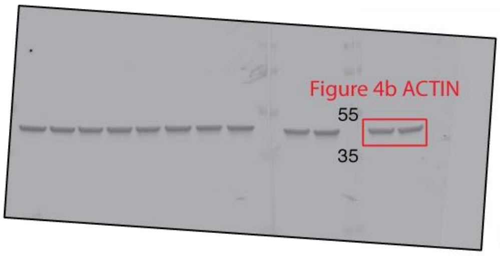
Figure 4b ACTIN
55
35

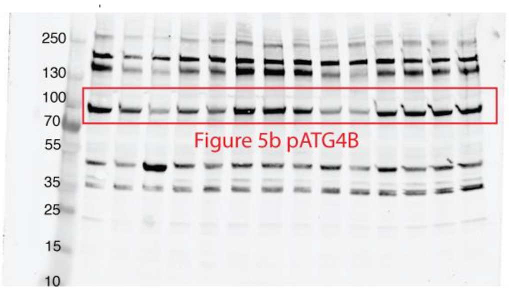
250
130
100
70
55
35
25
15
10
Figure 5b pATG4B

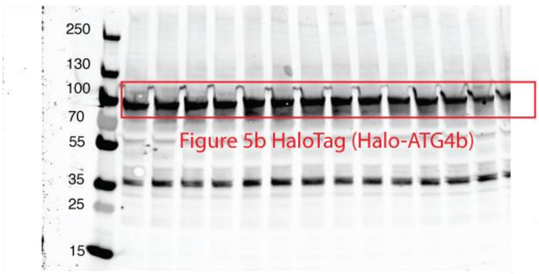
250
130
100
70
55
35
25
15
Figure 5b HaloTag (Halo-ATG4b)

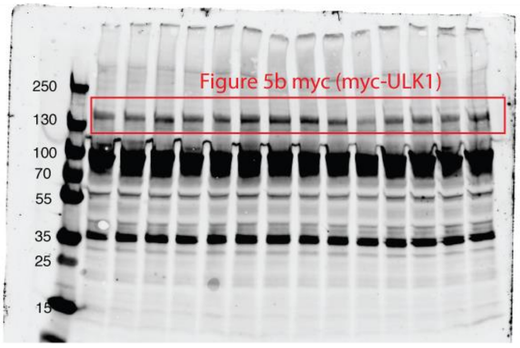
Figure 5b myc (myc-ULK1)
250
130
100
70
55
35
25
15

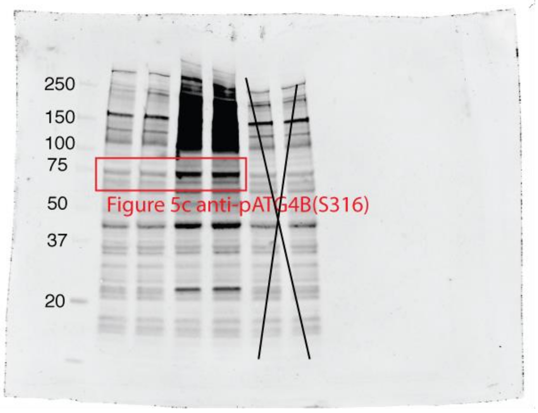
250
150
100
75
50
37
20
Figure 5c anti-pATG4B(S316)

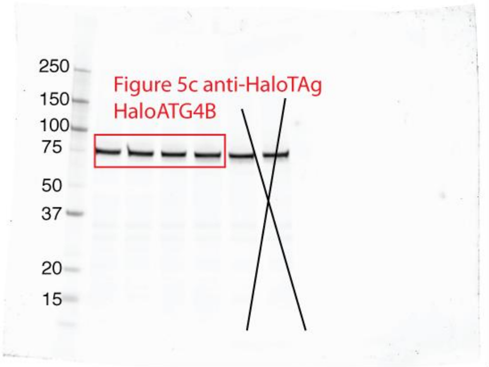
250
150
100
75
50
37
20
15
Figure 5c anti-HaloTAg
HaloATG4B

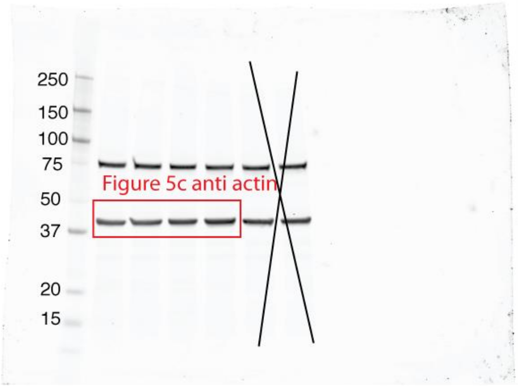
250
150
100
75
50
37
20
15
Figure 5c anti actin
